# Designing Magnetic Anisotropy through Strain Doping

**DOI:** 10.1002/advs.201800356

**Published:** 2018-10-10

**Authors:** Andreas Herklotz, Zheng Gai, Yogesh Sharma, Amanda Huon, Stefania F. Rus, Lu Sun, Jian Shen, Philip D. Rack, Thomas Z. Ward

**Affiliations:** ^1^ Materials Science and Technology Division Oak Ridge National Laboratory 1 Bethel Valley Rd. Oak Ridge TN 37831 USA; ^2^ Institute for Physics Martin‐Luther‐University Halle‐Wittenberg Halle 06120 Germany; ^3^ Center for Nanophase Materials Science Oak Ridge National Laboratory 1 Bethel Valley Rd. Oak Ridge TN 37831 USA; ^4^ Renewable Energies – Photovoltaics Laboratory National Institute for Research and Development in Electrochemistry and Condensed Matter Timisoara 300569 Romania; ^5^ SIST Shanghai Technology University Shanghai 200433 China; ^6^ Department of Physics Fudan University Shanghai 200433 China; ^7^ Materials Science and Engineering Department University of Tennessee Knoxville TN 37996 USA

**Keywords:** epitaxy, implantation, magnetism, spin orbit coupling, strain

## Abstract

The coupling between a material's lattice and its underlying spin state links structural deformation to magnetic properties; however, traditional strain engineering does not allow the continuous, post‐synthesis control of lattice symmetry needed to fully utilize this fundamental coupling in device design. Uniaxial lattice expansion induced by post‐synthesis low energy helium ion implantation is shown to provide a means of bypassing these limitations. Magnetocrystalline energy calculations can be used a priori to estimate the predictive design of a material's preferred magnetic spin orientation. The efficacy of this approach is experimentally confirmed in a spinel CoFe_2_O_4_ model system where the epitaxial film's magnetic easy axis is continuously manipulated between the out‐of‐plane (oop) and in‐plane (ip) directions as lattice tetragonality moves from ip to oop with increasing strain doping. Macroscopically gradual and microscopically abrupt changes to preferential spin orientation are demonstrated by combining ion irradiation with simple beam masking and lithographic procedures. The ability to design magnetic spin orientations across multiple length scales in a single crystal wafer using only crystal symmetry considerations provides a clear path toward the rational design of spin transfer, magnetoelectric, and skyrmion‐based applications where magnetocrystalline energy must be dictated across multiple length scales.

Control of spin orientation in crystalline materials is critical to fundamental and applied efforts related to magnetoelectrics,^1^ spin transport,^2^, ^3^ and skyrmion dynamics.^4^, ^5^ A common method of tuning spin orientation in crystalline materials is by exploiting magnetostriction and shape anisotropy effects. This is generally accomplished by imparting a particular strain state during the growth process through heteroepitaxy,^6^ thickness‐dependent strain relaxation,^7^ or shape anisotropy in nanocomposite structures.^8^, ^9^ A limitation in these methods is that it is impossible to manipulate magnetic anisotropy energies within the crystal post growth or to selectively change the local magnetic character within the as‐grown crystal due to the fact that these methods are limited to creating discrete, globally static strain fields. Recently, low energy He ion implantation was shown to be a viable means of strain doping epitaxial oxide films by inducing single‐axis out‐of‐plane (oop) lattice expansion while leaving the in‐plane (ip) axes epitaxially locked to the underlying substrate.^10^, ^11^, ^12^ In this work, we show that it is possible to continuously tune across a full range of magnetic anisotropies in epitaxial magnetic spinel films post growth. This is accomplished by exposing CoFe_2_O_4_ films to low energy He ion implantation which drives uniaxial oop lattice expansion, thereby changing the magnetoelastic energies of the film. This strain doping technique also allows for a range of coexisting anisotropies to be written into a single film by controlling where the He ions are directed, thus providing the ability to design multiple coexisting and varied magnetic properties across a range of length scales in a single film.

CoFe_2_O_4_ (CFO) is used as a model system in the present study. This ferrimagnetic spinel has a large magnetocrystalline anisotropy, which is chemically stable, and possesses nonlinear spin wave features that have made it of interest to multiferroic, magnetic storage, and spintronic applications.^13^, ^14^, ^15^ CFO's large magnetostrictive properties also mean that it is extremely tunable through modification of crystal shape, size, and strain state.^8^, ^16^ In this work, 30 nm thick CFO films are epitaxially grown on single‐crystalline (001)‐oriented MgO substrates using pulsed laser deposition. Implantation is done iteratively, which allows for comparative studies on a single film. **Figure** **1**A shows the *θ‐*2θ X‐ray diffraction (XRD) scans around the (004) peak of a CFO film before and after helium implantation at various doses. We observe the oop lattice parameter expands systematically with increased dose. While there is some peak broadening resulting from the slight variation of He distribution through the film thickness, the film shows no traces of impurity phases while Laue fringes confirm good film uniformity even at the highest dose. From the reciprocal space mapping (RSM) scan around the (103) MgO peak, we observe that the film remains epitaxially locked to the substrate across the entire doping range (Figure 1B). Peak fitting of the XRD and RSM scans are used to ascertain the ip and oop lattice parameters for each step in the dosing procedure. While the ip lattice parameter is shown to be *a*_film_ = *b*_film_ = 8.424 Å in all cases, the oop lattice parameter expands by >1.7% with *c*_film_ = 8.332 Å in the as‐grown state growing to *c*_film_ = 8.475 Å in the most heavily strain doped state of 3 × 10^16^ He cm^−2^. Defining tetragonality, as *t* = (*c*_film_ – *a*_film_)/*a*_film_, we see that strain doping is capable of providing a full and continuous range of tetragonal distortions in the same film: with tetragonality progressing from −1.1% in the as‐grown state to +0.6% in the highest strain doped state (Figure 1C). The ability to control tetragonality should have a profound impact on magnetic properties due to magnetostrictive effects.

**Figure 1:**
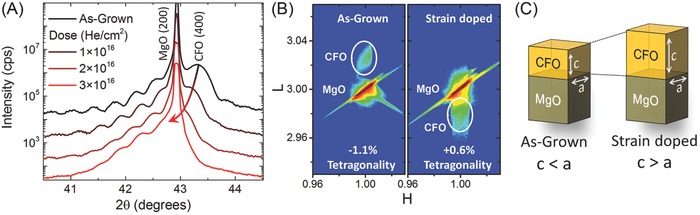
Implanting helium ions into a single crystal CoFe_2_O_4_ film grown epitaxially on MgO induces single‐axis oop lattice expansion. A)θ–2θ XRD around the (002)_pc_ MgO peak show that the *c*‐axis expands with increasing He dose (offset for clarity). B) RSM around the (103)_pc_ MgO peak for as‐grown and highest dosed states show that the film remains epitaxially locked to the substrate across the entire doping regime. C) Diagram illustrating change in tetragonality from negative value in as‐grown film where long axes lie in the film plane to a positive value where the long axis is directed oop.

By comparing the magnetization along the (010) ip and (001) oop directions of the same sample at 100 K after each implantation step, we observe the impact of uniaxial lattice expansion on magnetic properties. In the as‐grown film, an oop easy axis is clearly revealed by the larger oop magnetic remanence (M_R_) and coercive field (H_C_), which are consistent with previous reports on CFO thin films epitaxially grown on MgO^7^ (**Figure** **2**A and discussion in Supporting Information). The as‐grown ip magnetization loop was very soft with a saturation field near the limit of our SQUID magnetometer; as such, we note that the M_R_ and H_C_ may be slightly higher but only along the ip direction and only in the undoped system (see discussion in the Supporting Information). In the most highly expanded film, we see that the oop magnetic remanence and coercive field are both greatly reduced while the ip components increase, thus demonstrating an ip easy axis of magnetization (Figure 2B). We note that the narrowing of oop loop near zero‐field crossing and the relative difference in field direction response is nearly identical to previously reported behavior of pristine as‐grown tensilely strained CFO films having a tetragonality of +0.72%.^17^ The effects of successive increases to the c‐axis length on magnetic remanence and coercive field are shown in Figure 2C. The change in tetragonality is accompanied by a systematic reduction in the oop magnetic remanence and coercive field while increasing the ip components' values. The crossover point between easy axis direction can be identified by the region where the ip and oop values are equal. Interestingly, one might expect this crossover to occur at a tetragonality value of 0% which would indicate a cubic structure; however our observations are well below this—occurring at −0.38% for M_R_ and −0.15% for H_C_. This can be understood by considering that the total uniaxial anisotropy energy, *E*_a_, determines the direction of magnetic anisotropy and that the magnetoelastic energy driven by tetragonality is only one component of *E*_a_.^18^

**Figure 2:**
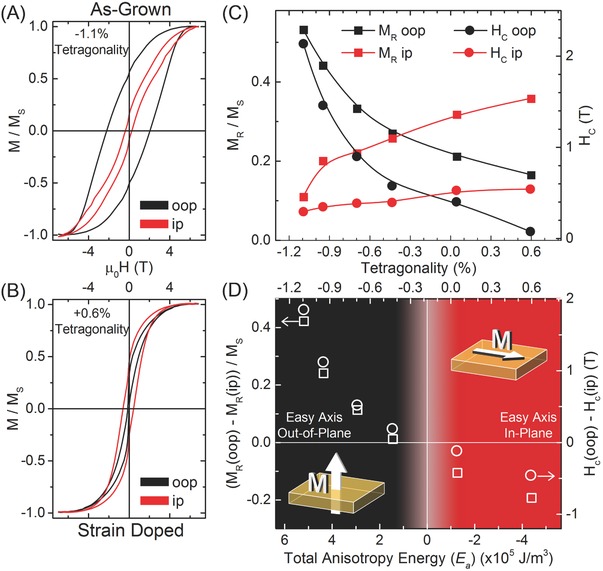
Effects of controlled single axis lattice expansion on magnetic properties. A) Magnetic hysteresis loops on the as‐grown film taken at 100 K with the magnetic field directed ip(red) and oop (black) show an oop easy axis of magnetization. B) Magnetic hysteresis loops of most highly strained doped sample. After the film is expanded to have a positive tetragonality, the easy axis is shown to lay ip. C) Comparison of magnetic remanence and coercive field values with field applied in‐plane and oop for the same sample after iterative strain doping. The crossover in easy axis direction can be recognized as the point at which the ip and oop values are equal. As the induced tetragonality shifts from negative to positive values, magnetic easy axis follows from oop to ip. D) The calculated total anisotropy energy of each strain state is compared to their measured difference in remanences and difference in coercive fields along the ip and oop directions.

For the (100) oriented CFO film of the thickness investigated in this work, we can estimate the total anisotropy energy as *E*_a_ = *K*_ME_ + *K*_Shape_, where the magnetoelastic component is given as *K*_ME_ = −3/2 λ_[100]_ (*c*_11_−*c*_12_) (ε_2_−ε_1_) and the shape contribution is given as *K*_Shape_ = −1 × 10^5^ J m^−3^ with surface contributions ignored due to the relatively large film thickness.^7^, ^18^, ^19^, ^20^ The magnetoelastic energy is determined using the bulk values of the elastic constants, *c*_11_ = 2.73 × 10^12^ dyne cm^−2^ and *c*_12_ = 1.06 × 10^12^ dyne cm^−2^, the magnetostriction constant along the [100] direction λ_[100]_ = −2.25 × 10^4^, and the difference between the oop strain, ε_2_ = (*c*_film_ − *c*_bulk_)/*c*_bulk_, and ip strain, ε_1_ = (*a*_film_ − *a*_bulk_)/*a*_bulk_.^20^ We note that the actual elastic constants may evolve under the induced volume increases, so thiscalculation is provided as a rough predictor. By calculating the expected anisotropy energy for each strain state and comparing it to the differences between the experimentally observed oop and ip magnetic remanences and coercive fields, we recognize the trend of oop easy axis of magnetization for *E*_a_ > 0, ip easy axis of magnetization for *E*_a_ < 0, and the spin reorientation transition occurring near *E*_a_ = 0 (Figure 2D). The effects of low energy He ion implantation on magnetic easy axis can thus be well described simply by estimating how the lattice symmetry influences the total anisotropy energy in the system. These results are fully consistent with previous works in which thickness‐dependent strain relaxation^7^ or shape anisotropy in nanocomposites^8^, ^9^ were used to compare how structural differences impact the spin reorientation transition. It is important to recognize that these previous approaches were based on comparing different samples and relied on generating different lattice environments between samples by changing sample thicknesses and growth conditions. The strain doping approach may thus allow for a cleaner investigation of structure–function relationships in fundamental studies, because it can provide iterative manipulation of structure in the same sample. The capability of fine tuning lattice symmetry post‐growth may also have importance to applications, as it enables the setting of very exact magnetoelastic anisotropies that can allow for extreme precision in designing structure‐driven functional properties.

Structure manipulation is not limited to whole film applications because the incoming He ions can be localized with masking. This opens the possibility of generating multiple coexisting strain states in the same crystalline sample. The simplest approach to creating multiple strain regions is to partially cover the sample with a thick mask during implantation, thereby creating two strain states in the same sample. However by extending this concept, we may generate a continuously varying strain from as‐grown to highly expanded across the entire sample surface. To do this, we place a linearly translatable in situ ion beam blocking mask directly over the sample and slowly retract the mask across ≈90% of the sample's surface during implantation (**Figure** **3**A). The nominal dose range across the sample surface then runs from as‐grown to 3 × 10^16^ He cm^−2^. The result is a single crystal material which contains a full and continuous shift in tetragonality across the 5 mm sample length. We refer to this process as gradient dosing and can observe its effects on local lattice symmetry with Raman spectroscopy (Supporting Information). Using a 50x objective on a confocal Raman Microscope, the five active modes expected by group theory analysis for the cubic spinel CFO with the space group of (Fd3 ®m)^21^ are locally probed. The equally spaced measurements from the undosed region toward the highest dosed region each exhibit a similar Raman spectral profile (Figure 3B). The active phonon modes undergo a continuous blue shift without a significant drop in relative intensity as a function of increasing He dose (Figure 3C). This indicates a smooth increase in the film's oop tetragonal distortion while signaling that the implantation process is not inducing significant structural disorder^22^, ^23^, ^24^—an important distinction from previous works that relied on disordering metallic films^25^, ^26^ and correlated oxides^27^, ^28^ using high energy and heavy noble elements to alter magnetic properties. Much like traditional strain engineering techniques, the low energy strain doping approach relies on modification of lattice symmetry to give control over magnetic anisotropy, making it far less susceptible to the reductions in magnetization inherent with the disordering approaches. At the same time, strain doping's access to iterative and continuous control over local and global anisotropy energies provides new opportunities to rationally design magnetic functionalities into crystalline materials that are not possible under the limitations imposed by traditional strain engineering methods that only permit discrete, global symmetries to be applied.

**Figure 3:**
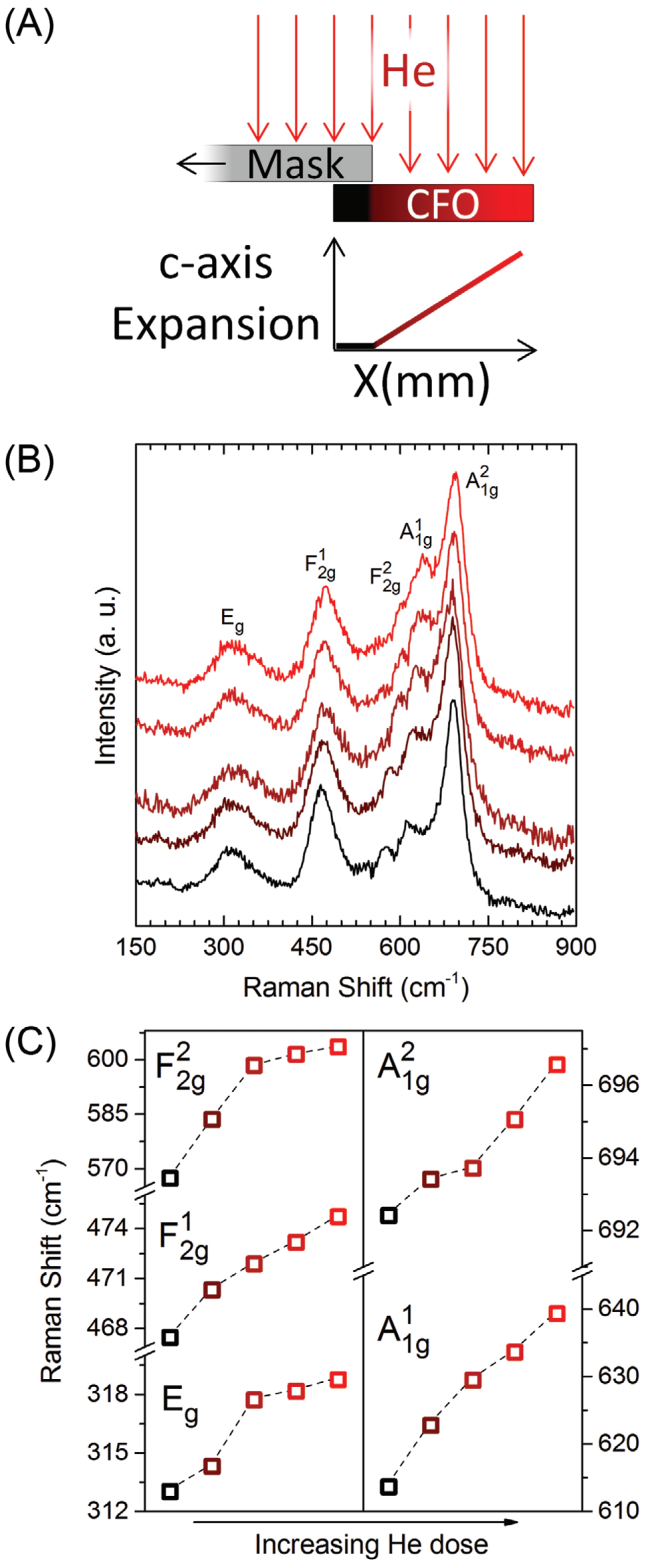
Gradient dosing approach. A) Conceptual diagram of method used to induce a continuous change in uniaxial expansion across the length of a sample. During the implantation process, an in situ ion beam blocking mask is slowly retracted across the surface of the CFO film which creates a gradient of strain states in the same film. B) Raman scans taken at different points along the film's length exhibit five active modes. C) These modes show a clear blue shift with increased He dose which is associated with oop lattice expansion.

Ion implantation allows for the application of lithographic techniques to generate local strain inhomogeneities (Figure S1, Supporting Information). **Figure** **4**A provides a diagram of how lithographic masking can be used in conjunction with strain gradient masking to produce a full range of tetragonally distorted islands surrounded by pristine crystal. Using magneto‐optic Kerr effect (MOKE) microscopy at different positions across the length of a CFO sample then allows one to observe magnetic contrast differences at separate strain states (Figure 4B). In the pristine undosed region, we see no evidence of contrast at any point during the magnetic field sweep. However, as we move toward the more heavily strained regions, we observe an increasingly strong contrast in the island regions near 0 T instigated by the variation in local spin orientations resulting from the magnetoelastically driven remanence values. These observations offer a clear proof‐of‐concept example of the efficacy of strain doping to design and implement very complex magnetic anisotropies within a single crystal (Figures S4–S6 and Movie S1 in Supporting Information).

**Figure 4:**
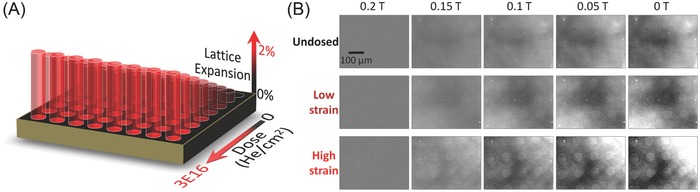
Lithographically defined masking combined with gradient dosing can be used to produce a range of magnetically defined textures in a single film. A) Defining a mask on the film surface allows local application of ion dose. Combining this with gradient dosing, a range of locally defined strain states can be applied in the same film to produce local islands of varied tetragonality surrounded by the unimplanted, as‐grown film. B) MOKE microscopy images taken with magnetic field applied oop at room temperature at different locations along the sample's length show that local magnetic states have been written into the film. The patterned islands are 50 µm in diameter. There is a clear contrast between the dosed regions and the surrounding as‐grown film as magnetic field is reduced due to the locally varied spin orientations dictated by the differences in magnetic remanences.

The ability to design local magnetic properties into single crystal films post growth opens many opportunities in applications related to magnetoelectrics and spin transport; and while the present work focuses on tuning magnetic susceptibilities by exploiting magnetostriction, electrostrictive materials should also offer many similar opportunities for designing local variations in materials' polar susceptibilities.^29^ The strong coupling between spin, charge, orbital, and lattice degrees of freedom in strongly correlated oxides means that very slight changes to lattice symmetry should also make it possible to tune these materials toward and away from functionally important phase boundaries.^10^, ^12^ Given the capability to manipulate these phases through lithographic methods, it should be possible to intentionally design locally dissimilar electronic and magnetic phase compositions across multiple length scales in single crystals using the existing ion implantation infrastructure currently used in the semiconductor industry. This would be an important step toward creating the single chip, multistimulus/multiresponse device architectures recently proposed for future “Beyond Moore” computing approaches in which a single chip combines various coexisting sensing and processing capabilities.^30^, ^31^

In summary, modification to lattice symmetry through low energy He ion implantation is a viable means to control magnetic easy axis of magnetization, which is demonstrated in CoFe_2_O_4_ films. This strain doping approach provides access to a continuous range of tetragonalities in an epitaxial system, which can then be used to modify a crystal's total anisotropy energy and magnetic properties. In situ ion beam blocking and/or lithographic techniques are shown to allow tetragonal distortions to be selectively applied to local and global length scales on the same sample. This approach allows iterative manipulation of structure which may provide a cleaner investigation of structure–function relationships in fundamental studies, while the capability of fine tuning lattice symmetry post growth may be critical to enabling the extreme precision required in designing structure‐driven functional properties. This demonstrates a clear path toward the rational design of spin transfer, magnetoelectric, and skyrmion‐based applications where spin orientations must necessarily be dictated across multiple length scales.

## Conflict of Interest

The authors declare no conflict of interest.

## Supporting information

Supplementary

Supplementary
