# Supplementary material for: Designing Magnetic Anisotropy through Strain Doping
Source: Adv Sci (Weinh). 2018 Oct 10;5(11):1800356. doi: 10.1002/advs.201800356 (PMC6247029; doi:10.1002/advs.201800356)
Supplement: Supplementary file 1 — Supplementary [file ADVS-5-1800356-s002.pdf]

Copyright WILEY-VCH Verlag GmbH & Co. KGaA, 69469 Weinheim, Germany, 2018.

ADVANCED SCIENCE
Open Access

Supporting Information

for *Adv. Sci.,* DOI: 10.1002/advs.201800356

Designing Magnetic Anisotropy through Strain Doping

*Andreas Herklotz, Zheng Gai, Yogesh Sharma, Amanda Huon, Stefania F. Rus, Lu Sun, Jian Shen, Philip D. Rack, and Thomas Z. Ward**

Supporting Information

Designing Magnetic Anisotropy through Strain Doping

*Andreas Herklotz, Zheng Gai, Yogesh Sharma, Amanda Huon, Stefania F. Rus, Lu Sun, Jian Shen, Philip D. Rack, and Thomas Z. Ward**

Sample preparation

Film growth

30 nm Epitaxial $CoFe_2O_4$ (CFO) thin films are grown on (001)-oriented MgO crystals by pulsed laser deposition (KrF 248 nm excimer laser). The growth temperature, oxygen pressure, and laser energy are 700°C, 40 mTorr and 1 J/cm^2, respectively.

Ion implantation

Before implantation, the films are covered with a 15 nm thick Au cap layer using RF sputtering. This cap acts to neutralize incoming ions, keep the sample from charging during implantation, and ensures that the CFO film is not sputtered. Helium is implanted using a SPECS IQE 11/35 ion source at an energy of 4 keV. From SRIM modelling, this energy and cap thickness puts the central peak of the implanted region near the middle of our 30 nm CFO films' thickness. SRIM is used as a rough estimate of resting location as it is based on the films being amorphous, thus the best measure of ion distribution is by observing post-irradiation structural data (**Fig. S1**). To study iterative strain accumulation effects, we remove the Au cap after each implantation cycle using mechanical polishing with a Kimwipe followed by rinses with methanol and water. After characterizations, a new Au cap layer is applied for the next implantation step. KI may also be used to remove the Au layer.

In figures 1 and 2 of the manuscript, we have applied this method by characterizing the as-grown state (*t*=-1.1%), and then using iterative dosing to reach the 5 strain doped tetragonalities (*t*) shown. The nominal dosages applied were 5E15(*t*=-0.9%), 1E16(*t*=-0.7%), 1.5E16(*t*=-0.4%), 2E16(*t*=+0.1%), and 3E16(*t*=+0.6%). This means the first dose was 5E15 He / cm^2, the second dose added 5E15 He / cm^2 which brought the total applied dose to 1E16 He / cm^2, the third dose added another 5E15 He / cm^2 which brought the total applied dose to 1.5E16 He / cm^2, the forth dose added 5E15 He / cm^2 which brought the total applied dose to 2E16 He / cm^2, and the final dose added 1E16 He / cm^2 to bring the total dose to 3E16 He / cm^2.

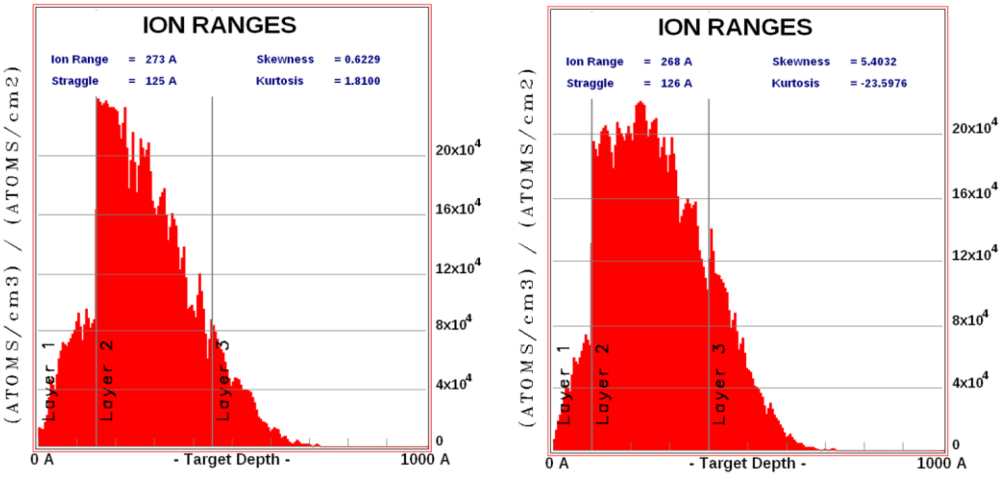

Figure S1. Examples of stopping and range of ions in matter (SRIM) simulated resting locations for 4kV He ion irradiation on two different Au cap layer thicknesses [15 nm (left) and 10 nm (right)] on 30nm amorphous $CoFe_2O_4$ on MgO substrate—Au is marked layer 1, CFO is marked layer 2, and MgO is marked layer 3.

Lithographic masking

Lithographic patterning is accomplished using standard UV photoresist as a blocking medium (**Figure S2**). As-grown single crystal films are spin coated with ~1 μm photoresist which is developed using a lithographic mask and UV light to form openings to the film. A thin Au cap layer is applied to the entire surface before He ion irradiation is applied. The photoresist and Au cap are removed with acetone and gentle mechanical polishing with a Kimwipe, which leaves a film with local lattice expansion resulting in a change of magnetic spin texture arising from magnetostrictive effects.

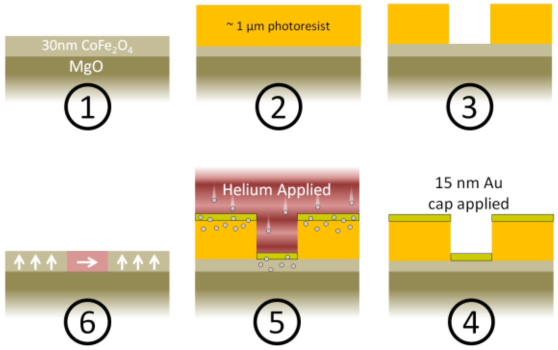

Figure S2. Lithographic steps used to generate locally implanted regions. The arrows in step 6 indicate the magnetization direction in the example CFO film.

Magnetometry

Magnetic hysteresis loops taken along the in-plane and out-of-plane directions were collected on a Quantum Design MPMS3. We have removed 2 data points from each out of plane MvH loop

direction where the raw data showed the SQUID undergoing a range change. These points were not near regions which would impact observation of coersive field, saturation magnetization, or magnetic remanence values. Room temperature magnetization measurements before and after a relatively high dose of 2E16 He/cm^2 are shown in **Fig. S3**. For as grown, the M_s is ~ 335 emu/cm^3 which is as expected for 30nm CFO on MgO. After dose sufficient to generate a tetragonality of 0.5%, we get M_s ~318 emu/cm^3. The change in magnetic remanence and coercive field are consistent with properties observed at lower temperatures—uniaxial lattice expansion moves the easy axis of magn3 etization from the out-of-plane to the in-plane direction.

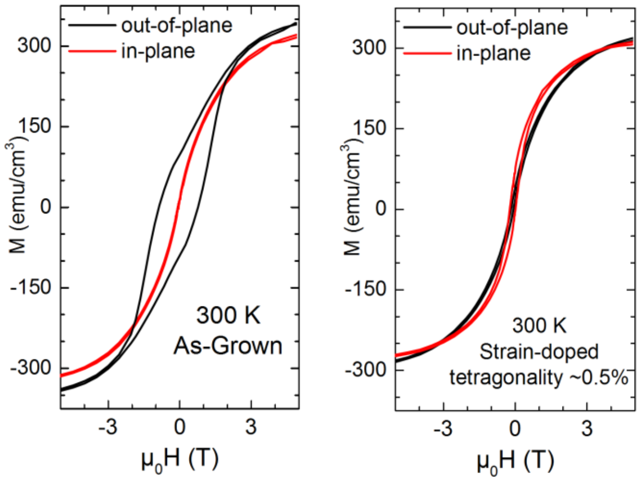

Figure S3: Room temperature comparison of magnetization loops before and after strain doping. We observe that the change in easy axis is consistent with low temperature magnetometry and room temperature MOKE observations. The difference in measured moment and coercive field between high and low temperature is well known and can be seen in references in the manuscript. The change in easy axis again fits our explanation of being driven by controlling lattice symmetry.

The magnetization loops used to locate M_R and H_C for each tetragonality are shown in **Figure S4a-b**. In both the in- and out-of-plane loops we see a smooth transition as uniaxial lattice expansion is applied. During review, a reviewer suggested that the in-plane as-grown loop may not have been fully saturated. The as-grown in-plane magnetic behavior is very soft so it is difficult to be completely certain. Figure S4c gives evidence for the loop likely being saturated. The divergence in slope is often used to signal the onset of saturation in soft magnets. Further the return loop at highest field has a magnetization that is measured as very slightly higher than the first data point on field reduction. The first point, while being at higher field, has a lower measured moment than the second point measured at lower field. This suggests that we are within the noise in regard to saturation. These data combined with the slope change is very strong evidence that saturation has been reached since this behavior would not be possible for a minor loop. Still, the actual value of M_R and H_C for the as-grown in-plane loop could be very slightly higher. This also means that the calculated differences in M_R and H_C for the as-grown

state could be slightly lower than those shown in Figure 2 of the manuscript. Importantly, these assertions in no way negate our observation that changing symmetry with strain doping enables the controllable modification of magnetic easy axis which can be predictively correlated to the calculated anisotropy energy.

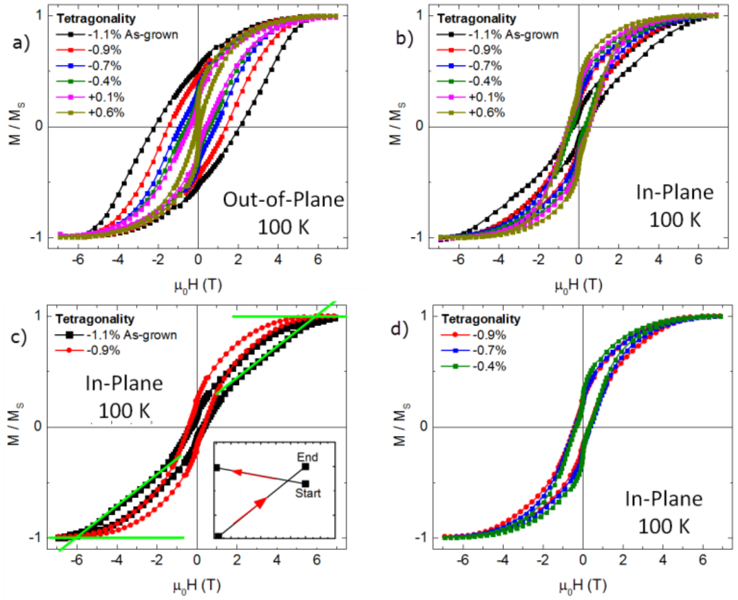

Figure S4: Comparison of all magnetization loops taken at 100 K for each of tetragonalities. a) out-of-plane and b) in-plane measurements show smooth shift in magnetic behavior as strain doping is applied. c) the in-plane as-grown loop is taken to be saturated due to the change in slope and the fact that the return magnetization at highest field is higher than the highest values taken at the start of the loop. d) the other 3 softest magnetic loops are closed.

Raman Spectroscopy

Raman scattering measurements were performed with a Renishaw 1000 confocal Raman microscope in back scattering configuration. The Raman spectra exhibit five active modes expected by group theory analysis for the cubic spinel CFO with the space group of $(Fd\bar{3}m)$ [21]. Each spectrum is a sum average of 5 individual spectra taken at different place on the sample through 50× objective with a 5 second acquisition time. The wavelength of the Raman laser used in these measurements was 532 nm. Raman studies are conducted with low laser power (2.5 mW) in order to avoid laser heating of the sample. In previous works, strong intensity drops in the active modes have been attributed to possible B-site disorder [23, 24]. In the present work, the relative peak intensities are not much affected by increased He dose though there is some broadening on all peaks. This broadening can be most easily attributed to a slight inhomogeneity of strain field through the film's thickness which results from the slight non-uniformity in He distribution. As an example, even at the highest dosing conditions, the A_{1g} modes do not significantly decrease, thus we infer that there is no substantial disordering occurring under the higher dosing conditions and that the blue-shift of the Raman modes indicates the dominant role of tetragonal distortion in our dosed CFO films. This shift was previously reported in

comparative strain relaxation studies and is related to a change in the out-of-plane tetragonal distortion [21]. In case of tetragonal distortion, one can expect a consistent blue-shift of the phonon modes as the effect of the expansion of the c-axis, which is the case in our CFO film in agreement with the x-ray results.

Gradient dosing and MOKE microscopy

The Magneto Optical Kerr Effect (MOKE) microscopy data presented in the manuscript is performed on a lithographically printed array of 50 μm diameter circular openings spaced 50 μm apart onto an undoped CFO film. The gradient dosing technique described in the manuscript is then applied. The photoresist and Au cap are removed with acetone and mechanical polishing with a Kimwipe. Optical microscopy images after cleaning show no signs of residual Au patterning.

Out-of-plane magnetic field: MOKE microscopy images were taken with polar sensitivity, by using a commercial Kerr microscope (Evico Magnetics). Out-of-plane magnetic field images presented in the main text were collected by applying a saturating magnetic field, focusing the microscope at a desired area, and taking snapshots as magnetic field was reduced toward 0T in 0.05 T steps. In all cases, the reference subtraction image is taken at 0.2 T magnetic field, 32 images are averaged at each set field, and image contrast is not adjusted. **Figure S4** includes images provided in the manuscript along with 2 additional intermediate regions. The black cloudy regions observed in the low field images is an experimental artifact arising from the Faraday Effect experienced at high fields—which is where the subtraction image is taken. Still we are able to see that the locally defined strained regions' intensities appear to change more significantly than the surrounding unexpanded region. This is consistent with the softening of the out-of-plane magnetic spin component.

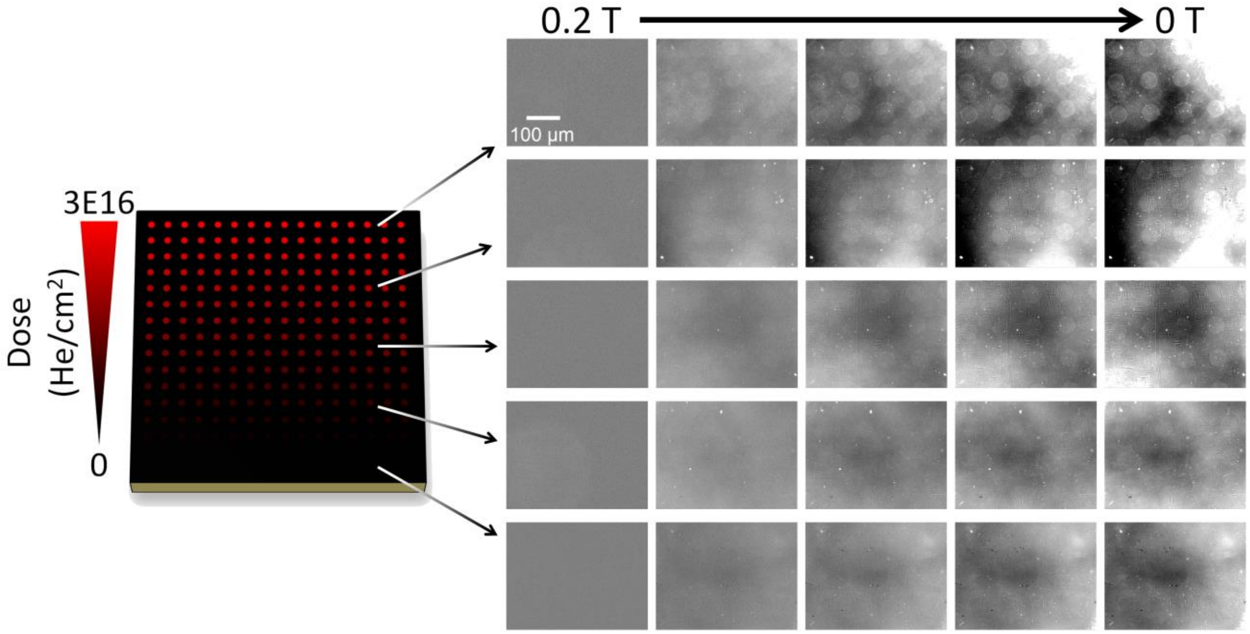

Figure S4. Schematic diagram of local strain array which gives relation to position of MOKE microscopy measurement locations. The locally defined regions range in tetragonality from -1.1% in the unimplanted state to ~+0.6% in the 3E16 He/cm^2 region.

In-plane magnetic field: MOKE microscopy using in-plane magnetic field was also collected on the sample described above. **Figure S5** shows MOKE microscopy taken at the center of the sample in the vicinity of what is referred to as the low strain region of figure 4 in the manuscript. In this field geometry, we are not significantly affected by the Faraday Effect though the absolute change in magnetic remanence along the in-plane direction is less than with the out-of-plane direction so the absolute contrast difference between strained and unstrained regions is reduced. Importantly, the locally defined strained regions' intensities change less significantly than the surrounding unexpanded region. This is consistent with a hardening of the in-plane magnetic spin component and is consistent with expectations from bulk magnetometry.

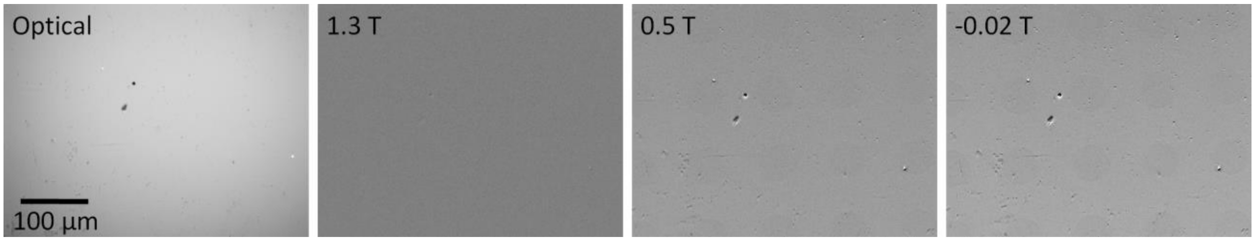

Figure S5. In-plane MOKE microscopy at center of the sample discussed above. Optical image is included to show that no Au pattern remains on the surface. The smaller relative intensity change of the strained regions as field is reduced toward 0T denotes the spin polarization staying oriented toward the initial in-plane set state more strongly than the surrounding region.

Similar experiments with in-plane field geometry were repeated on a 40 nm thick CFO film with various shapes lithographically applied to the surface and then uniformly implanted to create local regions of tetragonality of ~+0.5 % surrounded by pristine -1.1% tetragonality. **Figure S6** shows screen grabs from video collected during MOKE microscopy while focused on a locally expanded region with the magnetic field set to oscillate between ~±1.2 T. See full video in additional supplemental video. To allow full field sweeps without saturating the image at the largest positive and negative fields, we collect the reference subtraction image at 0 T and filter all collected images using that single image.

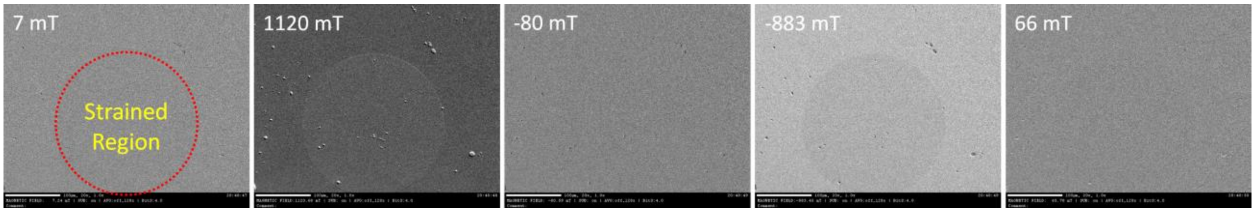

Figure S6. Images from video taken during field sweeps with magnetic field applied in-plane. Scale bar is 100 µm.

Movie S1

MOKE microscopy while focused on a locally expanded region with the magnetic field set to oscillate between ~±1200 mT. To allow full field sweeps without saturating the image at the largest positive and negative fields, we collect the reference subtraction image at 0 T and filter all collected images using that single image.
